# Factors affecting and effects of hemodynamic stability of pediatric patients with grades 3–5 renal trauma: a prospective non-randomized comparative study

**DOI:** 10.1186/s12894-023-01381-9

**Published:** 2023-12-08

**Authors:** Rabea Ahmed Gadelkareem, Ahmed Hamdan, Amr Abou Faddan, Hisham Mokhtar Hammouda, Mohamed Ali Zarzour

**Affiliations:** Assiut Urology and Nephrology Hospital, Faculty of Medicine, Assiut University, Elgamaa Street, Assiut, 71515 Egypt

**Keywords:** Conservative treatment, Kidney, Nephrectomy, Pediatrics, Shuttered kidney, Renal trauma

## Abstract

**Background:**

Researches on the effect of hemodynamic stabilization on the implantation of conservative management for pediatric high-grade renal traumas are lacking. We aimed to assess the effect of maintaining the initial hemodynamic stability of pediatric patients with grades 3–5 renal trauma on the implementation of the conservative treatment and identify its defining factors.

**Methods:**

A prospective study was performed on pediatric patients with grade 3–5 renal traumas who presented to our hospital during July 2020–June 2022. Hemodynamically stable patients were compared with the unstable patients for clinical characteristics, hemodynamic stabilization, and rates of success of conservative treatment.

**Results:**

Forty-three patients were studied, including 26 boys and 17 girls. Of them, 28 (65.1%) patients presented with hemodynamic stability and 15 (34.9%) patients were unstable. Overall, 32 (74.4%) patients achieved and/or maintained hemodynamic stability for conservative management. There was a significant difference in blood pressure level at presentation (p < 0.001). The improvement of the hemodynamic parameters was significant per group and, in comparison (p < 0.001). The size of hematoma was significantly smaller in patients with hemodynamic stability (p = 0.023). Despite the longer (p = 0.033) hospital stay with conservative management, the rates of blood transfusion (p = 0.597) and hospital stay (p = 0.785) were not significantly different between both groups. The rates of nephrectomy and mortality were 14% and 0%, respectively. Blood pressure was independently associated with the achievement of maintained hemodynamic stability for conservative management (p = 0.022).

**Conclusions:**

Hemodynamic stabilization seems to be effective and safe for implementing successful conservative management for pediatric patients with high-grade renal traumas. Blood pressure was the only independent factor of maintaining hemodynamic stability.

## Background

The rates of surgical intervention and complications are associated with the high grades of renal trauma in pediatrics [1, 2]. Recent studies advocate conservative management for high-grade renal traumas, enhancing the chances of renal preservation against nephrectomy [3–5]. However, the difficulty of decision-making and the preservation of a sufficient capacity of time for patient safety are major concerns [4]. In addition, there is no consensus among the different trauma guidelines on the indications for the implementation of conservative treatment. The Société Internationale d’Urologie guidelines recommend surgical exploration; the European Association of Urology guidelines recommend surgical exploration only in cases of vascular injuries; and the American Urological Association guidelines recommend the initial implementation of conservative management. Moreover, the role of initial hemodynamic stabilization has not been adequately described in these guidelines [6].

This study hypothesized that achieving and maintaining initial hemodynamic stability provides higher rates of implementation and success for conservative treatment. The aims were to assess the effect of maintaining hemodynamic stability during the first 24 h on the implementation of conservative management and its predictors in pediatric patients with grades 3–5 renal trauma. Hemodynamic stability was defined as the control, correction, or resolution of the clinical and laboratory manifestations of hemodynamic instability for 24 h. The primary outcomes were the rates of hemodynamic stability within the first 24 h and successful conservative management. However, the secondary outcomes were the rates of nephrectomy and mortality.

## Methods

A prospective study was conducted on pediatric patients with grade 3–5 renal traumas treated in our hospital during July 2020–June 2022. This study was approved by the ethical committee at our university. The inclusion criteria were patients aged < 18 years with renal trauma grades 3–5. Patients who had been explored by trauma surgeons without proper urological assessment, initially managed in another hospital before referral, or had trauma older than 24 h were excluded from the study.

The clinical workups included stabilizing and monitoring the vital signs (blood pressure, heart rate, and body temperature) and hematuria or perinephric hematoma with intravenous fluids (1–2 boluses of 20 mL/kg of crystalloid fluids) were administered before blood transfusion (packed red blood cells in a dose of 10 ml/kg up to 4 times till resuscitation was achieved). The initial laboratory workups included the hematocrit, hemoglobin levels, and serum creatinine. Hemodynamically stable patients, either from the start or after initial hemodynamic resuscitations, were managed by the conservative approach, while patients with persistent hemodynamic instability were managed by immediate surgical exploration.

In the conservative approach, patients were managed by observation and monitoring of their vital signs, hemodynamic status, and laboratory values. Further laboratory tests included urine analysis, random blood sugar, and blood gases when indicated. Medications and serial examination, testing, and reimaging were performed while the surgical team and the patient were ready for urgent potential surgical interventions. In the interventional approach, patients were managed by minimally invasive maneuvers or open surgery. These were the components of management in our hospital [7]. Our protocol for the evaluation of renal trauma included initial abdominal ultrasonography, as a screening tool for the trauma of the abdominal organs, including the kidneys. However, the abdominopelvic contrast-enhanced computed tomography (CECT) was routinely performed when the suspicion of renal trauma was high and to define the grade of the trauma. Grades of renal trauma were defined according to the classification of the American Association Society of Trauma based on the CECT findings [8].

According to their hemodynamic status at presentation, patients were classified into two groups. The first group included patients who were hemodynamically stable (Stable group), and the second group included patients who were hemodynamically unstable from the start (Unstable group). Respective to these groups, patients who were stable from the start (from the Stable group) and those who were stabilized and maintained (from the Unstable group) on hemodynamic stability for the first 24 h after trauma were allocated to receive conservative management. However, immediate surgical exploration with renorrhaphy, partial, or total nephrectomy was implemented to the unstable patients in both groups: First, patients from the Stable group who became unstable (instability after an initial stability). Second, patients from the Unstable group who failed to be stabilized or failed to maintain robust stabilization after resuscitation (persistent instability).

Regardless of the trauma grade, conservative treatment included complete bed rest, broad-spectrum antibiotic, hydration, analgesic, serial monitoring of the hemodynamics, vital signs, urine color, serial hemoglobin and hematocrit values, and reimaging in the form of serial abdominal ultrasonography follow-up for the size of hematoma in all cases. In addition, CECT was repeated at 3-month duration from the date of trauma in patients with hematoma seen by abdominal ultrasonography at 1-month follow-up. Patients stayed in the hospital for strict monitoring until there were stable vital signs, clear urine, regression or absence of perinephric hematoma. After discharge, each patient had a follow-up for three months. At each visit, a history of recurring hematuria, loin pain, or fever is taken with follow-up ultrasonography for tracing of the residual hematoma.

Postoperative follow-up was performed with strict observation of the vital signs. Further evaluations were carried out by abdominal ultrasonography and serial measurement of hemoglobin and hematocrit values up to discharge from the hospital.

### Statistical analysis

The statistical package for social sciences, version 20.0 (SPSS Inc., Chicago, Illinois, USA) was used to analyze the data. In descriptive analyses, continuous variables were presented as mean ± standard deviation or median and range. However, categorical variables were presented as the number and percentage of each category. The continuous variables were compared with Student t-test or Mann Whitney U test, according to data distribution. However, the categorical variables were compared with the chi-squared or Fisher’s exact test accordingly. A multivariate logistic regression was performed to assess the factors contributing to the maintenance of hemodynamic stability. Two-tailed p < 0.05 was considered as statistically significant.

## Results

**Fig. 1:**
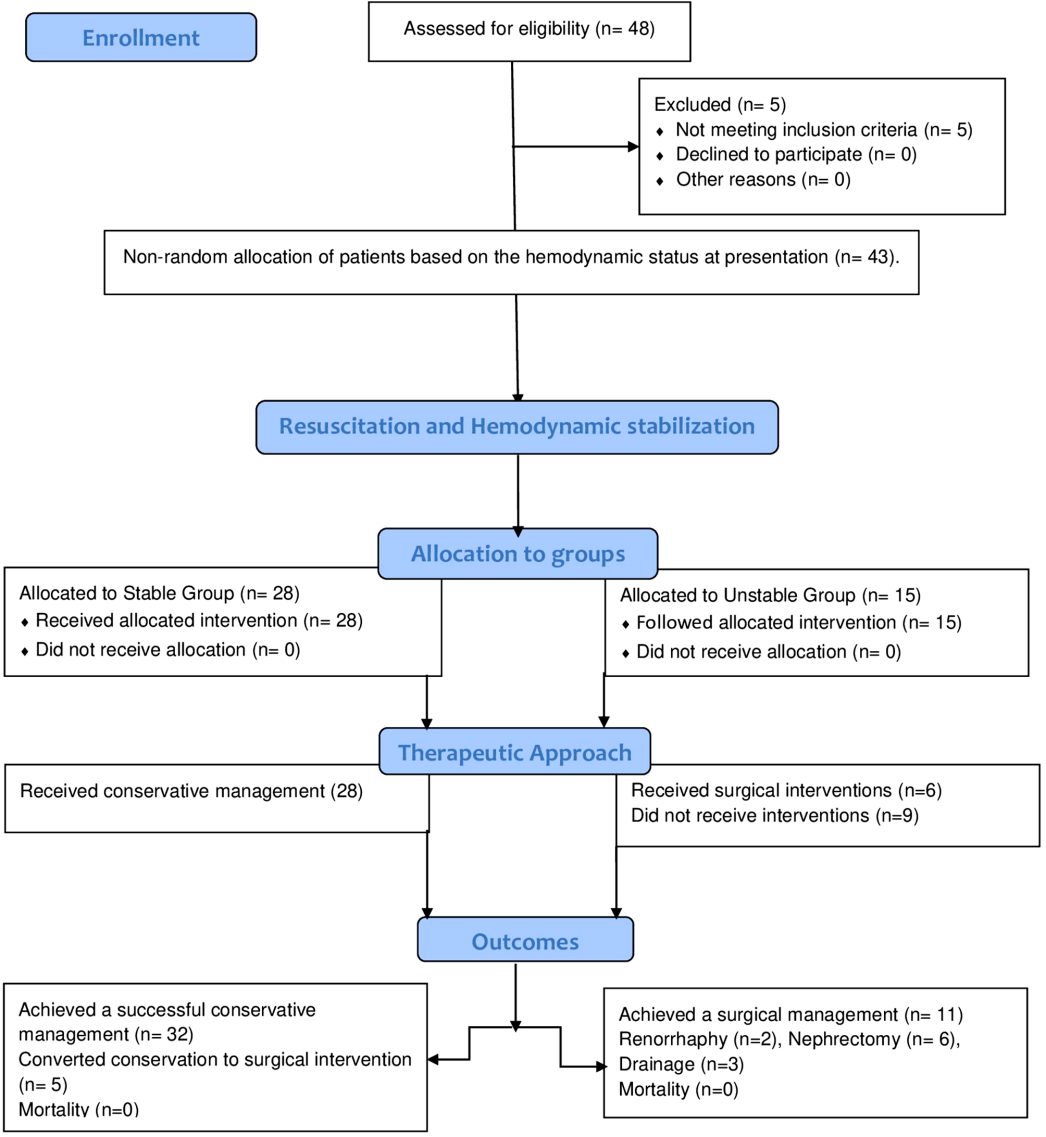
A flowchart of 48 pediatric patients who were treated in our center for high-grade renal traumas. They were differentiated into stable and unstable groups relative to their hemodynamic status and resuscitation at presentation through the first 24 h. Then, the stable patients received conservative treatment, but the unstable patients were treated by surgical interventions

During the duration of this study, 48 children were managed in the Trauma Unit, Assiut University Hospital, for grade 3–5 renal traumas. Of them, 5 patients did not fulfill the inclusion criteria and were excluded from the study; two patients were explored in another center before presentation to our hospital, and three patients were explored immediately with an inaccurate grading of renal trauma due to hemodynamic instability caused by multiple organ traumas (Fig. 1).

Forty-three eligible patients were included in the current study. Their mean age was 9.5 ± 4.6 years (Median = 10; range = 2–18 years) and mean body mass index was 24.4 ± 5.3 kg/m^2^. They included 26 boys (60.5%) and 17 girls (39.5%).

The first group included 28 (65.1%) patients with hemodynamic stability, and the second group included 15 (34.9%) patients with hemodynamic instability. The demographic and clinical characteristics of both groups at presentation to the emergency unit are presented in Table 1. There were no significant differences between the patients of both groups in the demographic and clinical characteristics.

**Table 1:** Demographic and clinical characteristics of patients in both groups

Variables		Stable group (N = 28)	Unstable group (N = 15)	p value
		Mean ± SD, Median (range) or Number (percentage)		
Age (years)		9.5 ± 4.4	9.7 ± 5	0.892
Gender	Male	17 (60.7%)	9 (60%)	0.964
	Female	11 (39.3%)	6 (40%)	
BMI (kg/m^2^)		24.4 ± 2.6	24.4 ± 2.5	0.951
Anatomical side of trauma	Right	13 (46.4%)	8 (53.3%)	0.666
	Left	15 (53.6%)	7 (46.7%)	
Etiological type of trauma	Animal kick	4 (14.3%)	3 (20%)	0.929
	Motor car accident	12 (42.9%)	7 (46.7%)	
	Fall from a height	12 (42.9%)	5 (33.3%)	
Degree of hematuria	Clear	14 (50%)	6 (40%)	0.122
	Mild	9 (32.1%)	3 (20%)	
	Moderate	3 (10.7%)	6 (40%)	
	Deep	2 (7.1%)	0 (0%)	
Blood pressure	Systolic	101.6 ± 5.8	88 ± 4.1	< 0.001
	Diastolic	70.2 ± 6.9	60 ± 11.2	0.001
Hemoglobin level		11.4 ± 1.2	10.7 ± 1.7	0.091
Hematocrit value		32.6 ± 4.2	30.9 ± 4.2	0.199
Serum creatinine		0.97 ± 0.25	0.97 ± 0.28	0.958
Perinephric hematoma by detection	Left side	11 (39.3%)	8 (53.3%)	0.556
	Right side	14 (50%)	7 (46.7%)	0.721
Grade of trauma by CECT	Grade 3	14 (50%)	3 (20%)	0.118
	Grade 4	9 (32.1%)	6 (40%)	
	Grade 5	5 (17.9%)	6 (40%)	
	Major vascular injuries^a^	2 (7.1%)	3 (20%)	0.211
	Size of perinephric hematoma (cm)	2.3 (0.2–9.3)	4.3 (1.5–7.5)	0.085

BMI: body mass index, CECT: contrast-enhanced computed tomography

^a^In the stable group, these injuries included injury of segmental arteries in the two cases. In the unstable group, however, they included thrombosis of the main renal artery in one case and injury of segmental arteries in two cases

During the first 24 h and through the whole first week after trauma, the changes in blood pressure were demonstrated (Table 2). Also, the means of daily hemoglobin and hematocrit values together with the means of the serum creatinine level up to patient discharge were monitored (Table 3).

**Table 2:** Means of blood pressure arterial pulse rate during the first 24 h and blood pressure through the first week in both groups*

Measure and timing		Stable group (N = 28)	Unstable group (N = 15)	p value**
		Mean ± Standard deviation		
At presentation	Systolic BP	101.61 ± 5.78	88.01 ± 4.14	< 0.001
	Diastolic BP	70.18 ± 6.87	60 ± 11.18	0.001
	Heart rate	103 ± 12	130 ± 18	< 0.001
After 1 day	Systolic BP	102.68 ± 6.31	91 ± 2.8	< 0.001
	Diastolic BP	71.79 ± 5.97	68 ± 6.49	0.061
	Heart rate	97 ± 11	113 ± 16	< 0.001
After 2 days	Systolic BP	105 ± 8.71	95 ± 11.5	0.003
	Diastolic BP	70.36 ± 5.6	65.67 ± 5.94	0.014
After 3 days	Systolic BP	105.18 ± 10.32	99.67 ± 13.69	0.144
	Diastolic BP	71.07 ± 5.33	67 ± 6.21	0.030
After 4 days	Systolic BP	117.32 ± 7.76	108 ± 4.93	< 0.001
	Diastolic BP	73.93 ± 5.33	66.67 ± 8.38	0.001
After 5 days	Systolic BP	112.5 ± 8.11	105.67 ± 7.04	0.009
	Diastolic BP	75.36 ± 6.6	70.33 ± 6.4	0.011
After 6 days	Systolic BP	113.39 ± 8.5	104.33 ± 5.63	0.001
	Diastolic BP	71.25 ± 15.07	65.8 ± 17.23	0.288
After 7 days	Systolic BP	117.32 ± 6.01	107.33 ± 5.63	< 0.001
	Diastolic BP	83.93 ± 5.33	77.73 ± 7.02	0.002
P value within group		< 0.001	< 0.001	

BP: Blood pressure (mmHg)

*These means of the heart rate (beats per minute) represent the statuses at presentation and during the whole first 24 h. **P value is significant if < 0.05

**Table 3:** Hemoglobin, hematocrit and creatinine levels in both groups

Variables		Stable group (N = 28)	Unstable group (N = 15)	P value
		Mean ± standard deviation		
**At presentation**	HB	11.41 ± 1.15	10.67 ± 1.65	0.091
	HCT	32.64 ± 4.18	30.86 ± 4.18	0.199
	SCr	0.971 ± 0.254	0.967 ± 0.276	0.958
**After 1st day**	HB	10.73 ± 1.43	10.52 ± 1.62	0.661
	HCT	31.09 ± 4.02	30.72 ± 2.80	0.750
	SCr	1.14 ± 0.360	1.1 ± 0.194	0.682
**After 2nd day**	HB	10.77 ± 1.48	10.52 ± 1.79	0.783
	HCT	30.82 ± 3.73	30.8 ± 2.22	0.990
	SCr	1.15 ± 0.315	1.1 ± 0.172	0.598
**After 3rd day**	HB	10.6 ± 1.28	10.78 ± 1.31	0.666
	HCT	30.92 ± 3.73	30.81 ± 2.22	0.925
	SCr	1.04 ± 0.287	1.01 ± 0.133	0.623
**After 4th day**	HB	10.27 ± 1.07	9.91 ± 1.31	0.331
	HCT	31.15 ± 3.85	31.05 ± 2.13	0.926
	SCr	1.11 ± 0.287	1.05 ± 0.172	0.412
**After 5th day**	HB	10.21 ± 1.08	9.87 ± 1.31	0.361
	HCT	33.53 ± 2.98	32.05 ± 2.78	0.160
	SCr	0.935 ± 0.261	0.853 ± 0.151	0.270
**After 6th day**	HB	10.51 ± 1.02	10.09 ± 1.16	0.230
	HCT	33.79 ± 3.14	32.44 ± 2.62	0.228
	SCr	0.906 ± 0.259	0.833 ± 0.140	0.313
**After 7th day**	HB	11.05 ± 0.911	10.79 ± 0.945	0.400
	HCT	35.23 ± 4.45	34.39 ± 2.95	0.543
	SCr	0.858 ± 0.279	0.805 ± 0.133	0.492
**P value within each group**	HB/HCT	0.002	0.001	
	SCr	0.001	0.012	

Abbreviations: HB; hemoglobin (g/dl), HCT; hematocrit, SCr; serum creatinine (mg/dl)

Of the 43 patients, 32 (74.4%) patients achieved and maintained hemodynamic stability in the first 24 h and they successfully completed conservative management. However, 11 (25.5%) patients were hemodynamically unstable or failed to maintain robust stability and they were managed by surgical interventions. In the unstable group, nine patients received successful conservative treatment. Among them, grades of trauma were 3, 4, and 5 in two, three, and four patients, respectively (Tables 4 and 5). Nephrectomy was performed in 6 patients (14%), but there was no mortality in this cohort of patients.

**Table 4:** Management and outcomes in the stable and unstable groups of patients

Variables		Stable group (N = 28)	Unstable group (N = 15)	p value
**Treatment success**				
Conservative treatment		23 (82.1%)	9 (60%)	0.119
G 3		14 (50%)	2 (13.3%)	
G 4		6 (21.4%)	3 (20%)	
G 5		3 (10.7%)	4 (26.7%)	
Surgical intervention		5 (17.9%)	6 (40%)	
G 3		0 (0%)	1 (16.7%)	
G 4		3 (10.7%)	3 (33.3%)	
G 5		2 (7.2%)	2 (50%)	
**Blood transfusion**				
Volume (units)		2 (1–4)	2 (1–3)	0.597
Patients		16 (57.1%)	15 (100%)	< 0.001
**Hematoma**				
Thickness of perinephric hematoma on discharge (cm)		1.1 (0–2.3)	2 (0.4–3)	0.023
Patients with residual hematoma	At 1 month	3 (13%)	4 (44.4%)	0.054
	At 3 months	0	0	
**Length of hospital stay** (days)		10 (4–30)	7 (4–21)	0.785

**Table 5:** Surgical interventions and their indications per hemodynamic status groups

Surgical procedure	Indications of intervention	Number of patients (%)	Hemodynamic stability group (number of patients)^a^
Nephrectomy	Exploration due to multiple traumas	3 (27.3%)	Unstable (2) Stable
	Persistent hematuria	2 (18.1%)	Unstable (2)
	Secondary hemorrhage	1 (9%)	Stable
Renorrhaphy	Persistent hematuria	1 (9%)	Unstable
	Increasing size of hematoma	1 (9%)	Unstable
Nephrostomy tube	Infected hematoma	1 (9%)	Stable
Double-J stent	Urine extravasation	2 (18.1%)	Stable (2)

^a^The hemodynamic status was similar to the main groups in the previous results

According to the two approaches of management, the grades of trauma and outcomes of management were compared. The rate of blood transfusion was significantly higher in the interventional group than in the group of conservative management (p < 0.001). However, the length of hospital stay was significantly longer with the conservative management (p = 0.033) than with the interventional management (Table 6).

**Table 6:** Summary of relevant variables to the approaches of treatment (grade of trauma, blood transfusion volume and length of hospital stay)^a^

Variables	Conservative group (N = 32)	Intervention group (N = 11)	p value
**Grade of trauma**			
Grade 3	16 (50%)	1 (9.1%)	0.057
Grade 4	9 (28.1%)	6 (54.5%)	
Grade 5	7 (21.9%)	4 (36.4%)	
**Blood transfusion**			
Patients	20 (62.5%)	11 (100%)	< 0.001
Volume (units)	2 (2–3)	3 (1–4)	
**Length of hospital stay (days)**	10 (10–12)	7 (6–21)	0.033

^a^This table is a summary of the treatment approaches and moat relevant variables. This presentation is not an alternative to the main design of the study and Results presented in other tables which were based on the classification of patients into stable and unstable groups

In a multivariate analysis, blood pressure was the only independent factor of maintaining hemodynamic stability for implementing conservative management (p = 0.022) (Table 7).

**Table 7:** Multivariate regression analysis to identify the factors affecting maintenance of hemodynamic stability during the first 24 h

Predictors	Odds Ratio	Standard Error	95% C.I.	p value
Age	0.163	0.017	0.056–0.701	0.274
Body mass index	0.428	1.295	0.034–5.414	0.512
Systolic blood pressure	0.770	0.009	0.230–1.068	< 0.001
Hemoglobin	0.040	0.087	0.016–0.196	0.858
Hematocrit	0.190	0.028	0.084–0.320	0.365
Higher injury grade (4 or 5)	0.154	1.677	0.006–4.109	0.264
No hematuria	0.847	1.844	0.023–31.442	0.928
Less blood transfusion unites	0.805	0.521	0.290–2.235	0.677

## Discussion

A large body of research has been conducted to verify the efficacy and safety of management approaches of high-grade pediatric renal traumas so far [3, 5, 9]. Despite this going on research, the conservative management still warrants a cautious application in practice because it may be confronted with the high variability of the definition of hemodynamic stability and the difficulty of decision-making in these high-grade traumas [2, 4]. In the current study, hemodynamic stability was defined based on the blood pressure and clinical responses to resuscitations. In addition, the conservative treatment warrants close monitoring and follow-up of patients, especially during the first few hours [4]. Hemodynamic stability is acknowledged as the most important factor in the assessment and management of blunt trauma patients. However, there is no consensus on the length of time during which the patient should be considered unstable and explored [2, 4]. We considered the trials of resuscitation successful when the patient could maintain stability for the first 24 h with no more than two trials of adjustment of blood pressure.

Furthermore, conservative management may be followed by a potential relative decline in renal function [4]. This effect warrants a long-term and accurate evaluation of the kidney by radioisotope studying [3]. The current study did not assess this long-term outcome because it targeted the stage of hemodynamic stabilization and its effect on the allocation to the management plan.

To make a decision about conservation in children with major renal trauma, there should be contingent safety and feasibility criteria available to guarantee timely intervention. In addition, this warrants the availability of a full range of flexibility in equipment and manpower for urgent exploration of the patient, whenever the indication for surgical intervention supervenes. The most critical period is the time from the presentation of the patient to the time of decision making, when the challenges of stabilizing a patient with a major hemorrhage are at their maximum [2, 4, 10]. In the current study, this part of management was achieved without threatening the lives of patients. Surgical intervention was timely for unstable patients who failed to achieve or maintain hemodynamic stabilization.

The mechanism of high-grade renal trauma is usually blunt injury, and grades 3 and 4 represent the highest proportion in those patients [3, 7]. In addition, hemodynamically stable patients with grade 5 renal trauma represent a rare event in high-grade renal trauma [11]. Similarly, the current results showed that all patients had blunt injuries, and grades 3 and 4 represented the highest proportions. In addition, more than 90% of patients in the unstable group had traumas of grade 4 or 5, while more than 50% in the stable group had traumas of grade 3. Hence, a success rate of 60% for conservative treatment in the unstable group may be an indicator of the favorable effect of successful initial resuscitation in these patients.

The recent studies showed high overall success rates for the conservative approach, reporting rates up to 92.5% [3.7]. The current results showed a slightly lower rate, but they are still relatively high in these grades of trauma in the context of the literature [2]. However, higher rates can be obtained if the issues of delayed referral and the uncertainty of the outcomes of these modern concepts of treatment have been resolved. Some patients may have a late presentation to our hospital due to the sequential referral protocol from the primary healthcare centers to the tertiary centers.

Extended renal investigations may not be required if the child’s hemodynamic instability persists or if there is no response to blood transfusions that are up to 3 units because immediate exploration is absolutely indicated. Expanding or pulsatile perirenal hematomas represent another indication for surgical therapy. In addition, the other common indications include significant urine extravasation, extensive (> 20%) nonviable tissue, arterial damage, and insufficient staging [12]. The surgical procedures and interventional techniques for management of high-grade renal traumas include renorrhaphy, partial nephrectomy, and nephrectomy. In cases of deeply lacerated kidneys without ischemic or completely separated parenchymal tissues, the collecting system should be sutured with absorbable monofilament sutures. In renorrhaphy, the margins of the lacerated parenchyma are reapproximated carefully, with or without interposing a hemostatic sponge or applying absorbable sealants to the suture. Omental flap interposition may be performed [13]. In the current study, renorrhaphy was performed in only two cases, as a simple approximation and suturing. Similarly, Ishida et al. reported no cases of renorrhaphy among 68 patients [14].

Partial nephrectomy is a higher step in the surgical repair of high-grade renal traumas with devitalized tissues. It is indicated in cases of a completely shattered pole of the kidney that is ischemic and its arterial supply is beyond repair. Early surgical debridement is the best treatment for devitalized renal parenchyma. Intraoperative signs of a devascularized pole or segment of the kidney include complete separation or bluish discoloration of the suspected tissues [13].

There are many indications for total or simple nephrectomy of the injured kidney. They include grade 5 injuries that are deemed irreparable, such as major vascular injuries, a shattered kidney, multiple concurrent injuries, and uncontrolled hemorrhage [2, 13]. Nephrectomy should be carried out if the diagnosis of renal artery thrombosis is postponed and laparotomy is otherwise necessary. If not, it may be decided to let the kidney atrophy and undergo a delayed nephrectomy if high blood pressure starts to appear [13].

The rate of nephrectomy is one of the main outcomes of the management of high-grade renal traumas. Compared to adult trauma, juvenile trauma has a lower nephrectomy rate [2]. It may be as low as 0% [15] or as variably high as 2.9–13% in some studies [14, 16, 17]. The current study showed that the rate of nephrectomy was high relative to the rate of repair, which may refer to the high potential of nephrectomy with surgical exploration. The rate of nephrectomy could be significantly reduced with the implementation of successful conservative management [2, 15]. Although the potential for mortality with high-grade renal trauma represents a significant risk [18], the mortality rate in the current study was at its minimum (0%).

The minimally invasive interventions in pediatric patients include angioembolization techniques, ureteral stents placement or percutaneous drainage of the obstructed kidneys [3–5, 19, 20]. The current study included ureteral stent and percutaneous nephrostomy placement in 3 cases only, representing minimally-invasive interventions.

As a prospective study, our study may contribute to filling the gap in research on the hemodynamic effect on management by providing information about the decision-making in the management of pediatric high-grade renal traumas. In addition, it allowed patient selection criteria that helped recruit the patients and manage them without harmful effects from the application of the conservative approach.

Limitations of his study included the non-randomized allocation of patients to the approach of management. The low incidence of high-grade renal trauma in pediatrics was a cause of the difficulty in recruiting a relatively larger sample size. In addition, this small sample size hindered studying the effect of hemodynamic stability on management in each grade of trauma. Furthermore, the short-term follow-up and evaluation of the kidneys did not allow us to know the extent of effect of trauma on the functions of the preserved kidneys after these high-grade traumas.

## Conclusions

In high percentages of patients presented with grades 3–5 renal trauma, achievement and maintenance of robust hemodynamic stability during the first 24 h were feasible, even in the unstable patients. It enhanced the implementation of conservative management and provided a high success rate and relatively low rates of nephrectomy and mortality. In addition, the blood pressure level at presentation was an independent factor in maintaining hemodynamic stability sufficient for successful conservative management in these patients.

## Data Availability

The data used and analyzed during the current study are available from the corresponding author on reasonable request.

## List of abbreviations

CECT: Contrast-enhanced computed tomography
